# Prostate-Specific Membrane Antigen-Based Therapeutics

**DOI:** 10.1155/2012/973820

**Published:** 2011-07-17

**Authors:** Naveed H. Akhtar, Orrin Pail, Ankeeta Saran, Lauren Tyrell, Scott T. Tagawa

**Affiliations:** ^1^Division of Hematology and Medical Oncology, Weill Cornell Medical College, 525 E. 68th Street, Box 403, New York, NY 10065, USA; ^2^Department of Urology, Weill Cornell Medical College, 525 E. 68th Street, Box 403, New York, NY 10065, USA

## Abstract

Prostate cancer (PC) is the most common noncutaneous malignancy affecting men in the US, leading to significant morbidity and mortality. While significant therapeutic advances have been made, available systemic therapeutic options are lacking. Prostate-specific membrane antigen (PSMA) is a highly-restricted prostate cell-surface antigen that may be targeted. While initial anti-PSMA monoclonal antibodies were suboptimal, the development of monoclonal antibodies such as J591 which are highly specific for the external domain of PSMA has allowed targeting of viable, intact prostate cancer cells. Radiolabeled J591 has demonstrated accurate and selective tumor targeting, safety, and efficacy. Ongoing studies using anti-PSMA radioimmunotherapy with ^177^Lu-J591 seek to improve the therapeutic profile, select optimal candidates with biomarkers, combine with chemotherapy, and prevent or delay the onset of metastatic disease for men with biochemical relapse. Anti-PSMA monoclonal antibody-drug conjugates have also been developed with completed and ongoing early-phase clinical trials. As PSMA is a selective antigen that is highly overexpressed in prostate cancer, anti-PSMA-based immunotherapy has also been studied and utilized in clinical trials.

## 1. Prostate-Specific Membrane Antigen

Prostate-specific membrane antigen (PSMA) is the single most well-established, highly specific prostate epithelial cell membrane antigen known [1–6]. The PSMA gene has been cloned, sequenced, and mapped to chromosome 11p [2, 7]. Pathology studies indicate that PSMA is expressed by virtually all prostate cancers [7–10]. Moreover, PSMA expression increases progressively in higher-grade cancers, metastatic disease and castration-resistant prostate cancer (CRPC) [3, 4, 11, 12]. Although first thought to be entirely prostate-specific [1–3], subsequent studies demonstrated that cells of the small intestine, proximal renal tubules, and salivary glands also express PSMA [5]. Importantly, the expression in normal cells is 100–1000-fold less than in prostate tissue [6], and the site of expression is not typically exposed to circulating intact antibodies [5]. In addition, PSMA is expressed on the neovasculature of the vast majority of solid tumor malignancies, but not on the normal vasculature [13]. In contrast to other well-known prostate-restricted molecules such as prostate-specific antigen (PSA) and prostatic acid phosphatase (PAP) that are secretory proteins, PSMA is an integral cell-surface membrane protein that is not secreted, thereby making PSMA an ideal target for monoclonal antibody (mAb) therapy. 

Prostate-specific membrane antigen has been found to have folate hydrolase and neurocarboxypeptidase activity [14]. Although its role in prostate cancer (PC) biology is unknown, the consistent finding of PSMA upregulation correlating with increased aggressiveness of the cancer implies that PSMA has a functional role in PC progression. Inhibition of enzymatic activity *in vitro* or in xenograft models has not demonstrated significant growth inhibitory effect (N. H. Bander et al., unpublished data). Nevertheless, the expression pattern of PSMA makes it an excellent target for mAb-based targeted therapy of PC.

Prostate-specific membrane antigen was initially validated as an *in vivo* target for imaging utilizing radiolabeled mAb 7E11 (CYT-356, capromab) [15, 16]. Capromab pendetide imaging was approved to evaluate the extent of disease in patients presenting with Gleason sums greater than 6 and those who experience a rising PSA after prostatectomy. Though improvements have been made with single-photon emission computed tomography (SPECT) and SPECT/CT imaging, because of suboptimal sensitivity and specificity of capromab pendetide, this imaging tool has not been widely adopted [17, 18]. Molecular mapping revealed that 7E11 targets a portion of the PSMA molecule that is within the cell's interior and not exposed on the outer cell surface [5, 19, 20] and cannot bind to viable cells [1, 20]. Recognition of these features by Bander and colleagues at Weill Cornell Medical College led to the development of mAbs to the exposed, extracellular domain of PSMA. In theory, the bound mAbs to the PSMA molecule would have the potential to significantly improve *in vivo* targeting and likely result in enhanced imaging and therapeutic benefit [20–22]. After testing, these antibodies (J591, J415, J533, and E99) did indeed demonstrate high-affinity binding to viable PSMA-expressing LNCaP cells in tissue culture and were rapidly internalized [20, 21]. Amongst these antibodies, the deimmunized IgG monoclonal antibody known as J591 was the most highly developed antibody clinically [23].

## 2. Radioimmunotherapy: Background and Rationale for Prostate Cancer

Radioimmunotherapy (RIT) is a technique by which a radionuclide is linked to a mAb or peptide and is typically delivered in a systemic fashion. In clinical practice, mAbs and peptides can be labeled with radionuclides that are usually beta-emitters. This “targeted” form of RT allows radiation delivery to tumors while sparing normal organs. The initially investigated form of RIT utilized radiolabeled antibodies against carcinoembryonic antigen for solid tumors. To date, the most studied form of RIT targets the CD20 antigen (^131^I tositumomab or ^90^Y ibritumomab tiuxetan) in non-Hodgkin's lymphoma, demonstrating safety and efficacy in phase I–III trials, which led to FDA approval. RIT for solid-tumor malignancies has been slower to develop. Reasons for this are multifaceted, including lack of specific antigens and antibodies optimized for RIT, difficulties in stably linking radionuclides to existing mAbs, shortfalls in existing (and readily available) radionuclides, and difficulty in clinical use (coordination between different specialties) [24]. However, clinical trials utilizing RIT in solid-tumor malignancies have been increasing.

The most common radionuclides employed have been ^90^Y and ^131^I, with ^177^Lu being used more recently. Based on the physical properties, each radionuclide may have an optimal tumor type and perform unique functions in clinical situations [25] (Table 1). 

Prostate cancer is an ideal solid tumor malignancy for the utilization of RIT; the tumor is radiosensitive with high exposure to circulating antibodies (bone marrow and lymph nodes) through typical distribution. Although sometimes clinically problematic, early readouts of efficacy can be examined using serum prostate-specific antigen (PSA) levels. In preclinical and clinical PC settings, radionuclides have been linked to antibodies and/or peptides against mucin, ganglioside (L6), Lewis Y (Le^y^), adenocarcinoma-associated antigens, and PSMA [26–36]. Of these, PSMA is the most specific and has been extensively studied in clinical trials.

Radioimmunotherapy can be delivered in a single dose or in multiple fractions. The degree of antitumor response following the administration of radiolabeled mAbs depends on several variables, specifically total (cumulative) radiation dose to the tumor, dose-rate, and tumor radiosensitivity. As with conventional external beam ionizing radiotherapy, dose fractionation may result in the ability to deliver a higher tumor dose with less toxicity. At the optimal dose-rate, fractionated dose RIT may decrease the amount of radiation to bone marrow while increasing the cumulative radiation dose to the tumor [37–39]. Preclinical data have shown that dose fractionation or multiple low-dose treatments can decrease toxicity while increasing the efficacy [40–42]. Early clinical studies have supported the ability to increase the cumulative maximum tolerated dose by dose fractionation [43–45].

Studies have shown that external beam RT can be combined with cytotoxic chemotherapy and, though toxicity may be increased, efficacy of concurrent chemoradiotherapy may be superior to sequential use. This may be especially true when utilizing chemotherapeutic agents with radiosensitizing effects. Combining RIT with cytotoxic chemotherapy has also been investigated [30, 31, 46]. These combinations have the possibility of increasing the therapeutic yield of RIT, particularly in the face of bulky, metastatic solid tumors.

With “targeted” therapy in general, patient selection can be significant. While our ability to preselect optimal PC patients based upon expression of a target has been limited, in other tumor types, reviewing targeted expression can be helpful in selecting patients more likely to respond or eliminating patients with a very low chance of response. For example, although epidermal growth factor receptor (EGFR) expression as measured by immunohistochemistry is not helpful in selecting patients for anti-EFGR mAb therapy in advanced colorectal carcinoma, excluding those with mutated K-ras has become helpful in clinical practice [47]. Specifically for PSMA expression, use of quantitative imaging, such as anti-PSMA-based positron emission tomography (PET) [48], may be more effective in selecting the best candidates (or ruling out poor candidates) for a PSMA-targeted therapeutic. When performing studies aimed to develop and examine predictive biomarkers, one must remember that prospective validation is important, as development of a “targeted” therapy may be thwarted by a suboptimal biomarker [49].

## 3. Anti-Prostatic-Specific Membrane Antigen-Based Radioimmunotherapy

Based on its apparent clinical ability to target some sites of disease, treatment studies were initiated utilizing radiolabeled capromab (CYT-356). In a phase I dose-escalation study, 12 patients with metastatic CRPC received ^90^Y-CYT-356 after biodistribution studies with ^111^In-CTY-356 [26]. As expected with RIT, myelosuppression was the dose limiting toxicity (DLT). No objective responses (PSA or radiographic) were noted. A subsequent phase II study utilizing ^90^Y-CYT-356 was performed in men with biochemically recurrent prostate cancer [30], yet the study was stopped after significant toxicity (myelosuppression) and lack of efficacy (no PSA decline) were seen in the first 8 patients.

**Figure 1:**
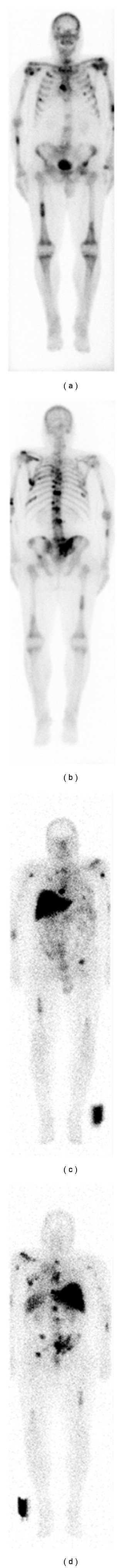
Radiolabeled J591 imaging. Left panels: Anterior (a) and posterior (b) images of pretreatment bony metastases on ^99m^Tc-MDP bone scan. Right panels: Anterior (c) and posterior (d) total body images obtained via dual-head gamma camera of sites of uptake 7 days after ^177^Lu-J591 administration. (note, antibody is partly cleared via the liver resulting in nonspecific ^177^Lu localization).

After determining that capromab was not capable of binding to viable PC cells, phase I clinical trials were performed linking Yttrium-90 (^90^Y) or Lutetium-177 (^177^Lu) to J591 via a DOTA chelate in patients with metastatic CRPC [25, 37]. Each of these studies was designed to deliver a single-dose of radiolabeled J591 intravenously followed by planar gamma camera imaging ± SPECT (in the case of ^90^Y-J591, imaging was performed after ^111^In-J591 administration). These trials defined the DLT and maximum tolerated dose (MTD) and further refined dosimetry, pharmacokinetics, and HAHA of the radiolabeled mAb conjugates and demonstrated preliminary evidence of antitumor activity. The vast majority of patients demonstrated good tumor targeting by radiolabeled J591. A representative planar gamma camera image of radiolabeled J591 is displayed in Figure 1. As expected, based on the physical properties as described above, the MTD of single-dose ^177^Lu-J591 was higher (70 mCi/m^2^) than that of ^90^Y-J591 (17.5 mCi/m^2^) [34, 35]. 

A phase II study was subsequently performed with ^177^Lu-J591, confirming safety, efficacy, and tumor-targeting ability [50]. In a dual-center study, men with progressive metastatic CRPC received a ^177^Lu-J591 intravenously followed by gamma camera imaging one week later. The results are promising and majority of patients demonstrated accurate targeting of known sites of metastatic disease, and PSA declines. All subjects experienced reversible hematologic toxicity without significant hemorrhagic complications. No serious drug-related nonhematologic toxicity occurred in either cohort.

In aggregate, based on the phase I and phase II data, these trials provide support that radiolabeled J591 is well tolerated with reversible myelosuppression, accurately targets PC metastatic sites, has antitumor activity, and is nonimmunogenic. However, as previously discussed, there are limitations to RIT for solid tumors, and the physical properties of ^177^Lu should be suboptimal in treating the population treated to date (men with progressive metastatic CRPC were treated, many of whom had bulky disease). Additional studies to improve the therapeutic profile are in progress.

Based upon the rationale above, a US Department of Defense sponsored study utilizing fractionated dose ^177^Lu-J591 has recently been completed with initial results of the primary endpoint presented [51]. Men with progressive metastatic CRPC received 2 fractionated doses two weeks apart. Doses were escalated in cohorts of 3–6 subjects, with cohort 1 receiving 20 mCi/m^2^ x2 and each successive cohort undergoing dose escalation by 5 mCi/m^2^ per dose (10 mCi/m^2^ cumulative dose increase per cohort). The primary endpoint was to determine DLT and the cumulative MTD of fractionated ^177^Lu-J591 RIT with pharmacokinetics and dosimetry, and the secondary endpoint was efficacy. Dose limiting toxicity was defined as severe thrombocytopenia (platelet count <15 or need for >3 platelet transfusions in 30 days), grade 4 neutropenia >7 days, febrile neutropenia, or grade >2 nonhematologic toxicity. Twenty-eight subjects received treatment with cumulative doses of up to 90 mCi/m^2^ (highest planned dose). The median age was 72 years with median baseline PSA of 49 ng/mL; the majority of subjects had Eastern Cooperative Oncology Group (ECOG) performance status of 1 and had bone metastases. The study confirmed the hypothesis that fractionated dosing would allow higher cumulative doses of ^177^Lu-J591 be administered with less toxicity.

Following progression on primary hormonal therapy, chemotherapy can offer symptomatic improvement as well as incremental survival benefit [52, 53]. However, responses are transient and all men eventually suffer from progression of disease as described above with single-agent anti-PSMA based RIT. The combination of taxane chemotherapy with RT has been used in several diseases because of the radiosensitizing effects of taxane-based chemotherapy [54–56]. In addition to favorable results from fractionated RIT and the radiosensitizing effects of taxane-based chemotherapy, it is hypothesized that the additional debulking by chemotherapy will overcome some of the limits imposed by the physical characteristics of ^177^Lu. Based upon this theory, a phase I trial of docetaxel and prednisone with escalating doses of fractionated ^177^Lu-J591 is ongoing [57]. 

As discussed above, the most studied form of RIT to date targets the CD 20 antigen (^131^I tositumomab and ^90^Y ibritumomab tiuxetan) in non-Hodgkin's lymphoma. While approved in the relapsed setting, it appears that these therapies have their greatest impact in the minimal disease setting [58–63]. The vast majority of relapses after local therapy for PC are initially “biochemical” only, that is, with a rising PSA despite no evidence of cancer on imaging, affecting approximately 50,000 men per year in the United States alone [64, 65]. Although there is no proven overall survival benefit in a prospective randomized trial, radiotherapy as a salvage regimen can lead to long-term survival in selected individuals [66–69]. Unfortunately, most individuals subsequently suffer systemic progression because of subclinical micrometastatic disease outside of the radiation field. 

Based on the demonstrated ability of J591-based therapy to successfully target known sites of disease and apparent clinical efficacy in the advanced setting, it is now under investigation in the salvage setting (clinicaltrials.gov NCT00859781). “Targeted radiotherapy” in the form of RIT is an attractive option with the possibility being a higher yield therapy in the minimal disease (biochemical only) setting. The primary objective of this trial is to prevent or delay radiographically evident metastatic disease. Radiolabeled J591 imaging will also be explored as a possible way to detect sites of disease in those patients with biochemical relapse and no evidence of disease on standard scans (^99m^Tc-MDP bone scans and computed tomography or magnetic resonance imaging) [70]. 

## 4. Anti-Prostatic-Specific Membrane Antigen Antibody-Drug Conjugates

Rather than linking a radionuclide to an mAb, a drug or toxin can also be linked, forming an antibody-drug conjugate (ADC) [71]. In this form of therapy, drugs may be delivered to target cells, sparing normal cells from toxicity. Many advances have been made in ADC technology. Gemtuzumab ozogamicin is an anti-CD33 mAb conjugated to calicheamicin which was approved by the US FDA in 2000 for older patients with relapsed acute myeloid leukemia, though it has recently been withdrawn from the market. Many others are in late-stage development, including trastuzumab-DM1 (anti-Her2 for breast cancer), inotuzumab, ozogamicin (anti-CD22 for non-Hodgkin's lymphoma), and brentuximab vedotin (anti-CD30 for Hodgkin's lymphoma).

MLN2704 is an ADC with maytansinoid 1 (DM1), which is a potent microtubule-depolymerizing compound conjugated to J591. Preclinical activity with MLN2704 was demonstrated [72] leading to a phase I trial designed to explore single ascending doses of the conjugate to define DLT, MTD, and PK [73]. Twenty-three subjects with metastatic CRPC received MLN2704 at doses ranging from 18–343 mg/m^2^ in an accelerated dose escalation scheme; 18 received at least 3 doses. Grade >3 toxicities occurred in 2 subjects, including 1 episode of uncomplicated febrile neutropenia and transient grade 3 elevation of transaminases. One subject (treated at 343 mg/m^2^) achieved a >50% decline in PSA, and another (treated at 264 mg/m^2^) experienced a PR by RECIST along with a >50% decline in PSA.

A subsequent multicenter phase I/II study was initiated based on the above results [71]. Sixty-two subjects received multiple doses of MLN2704. Four regimens were tested, with PSA declines most frequent at 330 mg/m^2^ every 2 weeks (2/6 had PSA decrease >50%, 2/6 had PSA stabilization). Although response was modest, and treatment was limited by toxicity, utilizing a PSMA mAb may be delivered in the clinic, and work is in progress and work is in progress utilizing new linkers to J591 designed to improve selective targeting. 

Based on the PSMA selective expression in PC and the principle above, additional researchers have initiated further clinical work with toxin-conjugates that target PSMA. In the preclinical setting, A5-PE40 and D7-PE40 are recombinant anti-PSMA immunotoxins tested *in vivo*. Huang et al. inhibited the tumor growth in mice bearing subcutaneous LNCaP tumors with an immunotoxin consisting of the anti-PSMA mAb E6 and deglycosylated ricin A [74]. Russell et al. coupled the melittin-like peptide 101 to anti-PSMA mAb J591 and obtained a significant tumor growth inhibition in mice [75]. Henry et al. used MLN2704 for the treatment of CWR22 xenografts [76]. Elsässer-Beile et al. reviewed other targeted systems against PSMA including RNA-aptamer-based immunotoxins [77]. Preclinical activity has been demonstrated in another mAb conjugated to monomethylauristatin E (MMAE) that recognizes the external domain of PSMA [78]. This work has led to a phase I dose-escalation study that has shown to be tolerated at the initial dose levels [79]. Additional early-stage clinical work has involved utilizing enzymatic activation to release cytotoxic substances in PSMA positive cells [80].

## 5. Anti-Prostatic-Specific Membrane Antigen Immunotherapy

Immunotherapy has been utilized in oncology over many decades, but only relatively recently has an autologous cellular immunotherapy agent (sipuleucel-T) been approved for clinical use in prostate cancer [81]. Though many attempts at utilizing immunotherapy in PC have focused on PSA [82, 83], as discussed, PSMA is an attractive target based on its restricted sites of expression. Multiple vaccine approaches have been utilized in preclinical models and have moved to early-stage clinical trials [83–88]. 

In addition to the deimmunization process in the transition from murine to human antibody, mAb J591 was engineered to interact with human immune effector cells and trigger antibody-dependent cell-mediated cytotoxicity (ADCC). In some of the initial studies with “cold” or “naked” J591 (unconjugated J591 with or without small doses of trace-labeled ^111^In-J591 for imaging purposes), stabilization of previously rising PSA occurred [89, 90]. Evidence of a dose-response relationship between mAb mass delivered and induction of ADCC was observed in a dose-escalation study enrolling patients with progressive CRPC [91]. One patient who received 100 mg of J591 had a >50% reduction in PSA.

Interleukin 2 (IL-2) promotes the proliferation and enhances the secretory capacity of all major types of lymphocytes, including T, B, and NK cells [92]. In addition, through its effects on NK cells, IL-2 stimulates antigen-nonspecific host reactions that involve interplay between NK cells and monocytes. Based on these functions, IL-2 may be useful as an immune stimulant, particularly in the setting of cancer immunotherapy [93]. Within two weeks of low-dose IL-2 treatment, selective expansion of human CD3^−^, CD56^+^ NK cells was seen with a plateau after 4 to 6 weeks of therapy [94, 95]. Based on the hypothesis that J591 plus IL-2 would work together to effect a positive immune response against prostate cancer, a combination study was initiated [96]. Seventeen patients with recurrent prostate cancer received continuous low-dose subcutaneous IL-2 (1.2 × 10^6^ IU/m^2^/day) daily for 8 weeks with weekly intravenous infusions of J591 (25 mg/m^2^) during weeks 4–6. Therapy was well tolerated with a trend for those with significant NK cell expansion to be nonprogressors.

## 6. Conclusion

In summary, PSMA is the most highly specific PC cell-surface protein known. Prostate cancer represents an ideal disease for mAb-directed therapy, with PSMA as an optimal target. Current strategies aim to improve upon past successes in utilizing anti-PSMA mAbs to deliver toxic payloads specifically to PC cells, minimizing damage to normal organs. Clinical use to date include developments with anti-PSMA RIT and ADC. Additional work in early stages of development includes anti-PSMA vaccines and utilizing PSMA-targeted therapy with or without other immune modulators to stimulate anti-PSMA ADCC.

##  Funding

Prostate Cancer Foundation, National Institutes of Health (ULI RR024996, 1-KL2-RR024997-01, PTBF5405), and Department of Defense supported this paper.

##  Disclosure

S. T. Tagawa is a member of the speakers bureau of Sanofi Aventis and accepted honoraria for lectures on docetaxel for prostate cancer within the past 3 years.

## Figures and Tables

**Table 1:** Radionuclide properties.

Radionuclide properties	^131^I	^90^Y	^177^Lu
Physical half-life (days)	8.05	2.67	6.7
Beta particles (mEv)			
max	0.61	2.280	0.497
average	0.20	0.935	0.149
Range in tissue (mm)			
max	2.4	12.0	2.20
average	0.4	2.7	0.25
Gamma emission (mEv)	0.364	None	0.113–0.208
Optimal size of tumor when targeted for curability [25]	3–5 mm	28–42 mm	1–3 mm
Comments	Cannot be used with internalizing mAb's	Lacks gamma emissions (cannot use for imaging)	
